# Disposition of Phytocannabinoids, Their Acidic Precursors and Their Metabolites in Biological Matrices of Healthy Individuals Treated with Vaporized Medical Cannabis

**DOI:** 10.3390/ph14010059

**Published:** 2021-01-13

**Authors:** Francesco Paolo Busardò, Ana Pilar Pérez-Acevedo, Roberta Pacifici, Giulio Mannocchi, Massimo Gottardi, Esther Papaseit, Clara Pérez-Mañá, Soraya Martin, Lourdes Poyatos, Simona Pichini, Magí Farré

**Affiliations:** 1 Department of Excellence-Biomedical Sciences and Public Health, Università Politecnica delle Marche, 60121 Ancona, Italy; fra.busardo@libero.it; 2 Clinical Pharmacology Unit, Hospital Universitari Germans Trias i Pujol and Institut de Recerca Germans Trias i Pujol (HUGTiP-IGTP), 08916 Badalona, Spain; appereza.germanstrias@gencat.cat (A.P.P.-A.); epapaseit.germanstrias@gencat.cat (E.P.); cperezm.mn.ics@gencat.cat (C.P.-M.); smartins.mn.ics@gencat.cat (S.M.); lpoyatos@igtp.cat (L.P.); mfarre.germanstrias@gencat.cat (M.F.); 3 Department of Pharmacology, Therapeutics and Toxicology, Universitat Autònoma de Barcelona, 08193 Cerdanyola del Vallés, Spain; 4 National Centre on Addiction and Doping, Istituto Superiore di Sanità, 00161 Rome, Italy; roberta.pacifici@iss.it; 5 School of Law, University of Camerino, 62032 Camerino, Italy; giulio.mannocchi@unicam.it; 6 Comedical S.Rl, 38123 Trento, Italy; massimo.gottardi@comedical.biz

**Keywords:** cannabis, cannabidiol, cannabidiol metabolism, medical cannabis

## Abstract

Inhalation by vaporization is a useful application mode for medical cannabis. In this study, we present the disposition of Δ9-tetrahydrocannabinol (THC), cannabidiol (CBD), their acidic precursors, and their metabolites in serum, oral fluid, and urine together with the acute pharmacological effects in 14 healthy individuals treated with vaporized medical cannabis. THC and CBD peaked firstly in serum and then in oral fluid, with higher concentrations in the first biological matrices and consequent higher area under the curve AUCs. Acidic precursors Δ-9-tetrahydrocannabinolic acid A (THCA) and cannabidiolic acid (CBDA) showed a similar time course profile but lower concentrations due to the fact that vaporization partly decarboxylated these compounds. All THC and CBD metabolites showed a later onset with respect to the parent compounds in the absorption phase and a slower decrease to baseline. In agreement with serum kinetics, THC-COOH-GLUC and 7-COOH-CBD were the significantly most excreted THC and CBD metabolites. The administration of vaporized medical cannabis induced prototypical effects associated with the administration of cannabis or THC in humans, with a kinetic trend overlapping that of parent compounds and metabolites in serum. The pharmacokinetics of cannabinoids, their precursors, and their metabolites in biological fluids of individuals treated with vaporized medical cannabis preparations showed a high interindividual variability as in the case of oral medical cannabis decoction and oil. Inhaled medical cannabis was absorbed into the organism earlier than decoction and oil. Cannabinoids reached higher systemic concentrations, also due to the fact that the acid precursors decarboxylated to parent cannabinoids at high temperatures, and consequently, the physiological and subjective effects occurred earlier and resulted with higher intensity. No serious adverse effects were observed.

## 1. Introduction

Medical cannabis is formed by Δ9-tetrahydrocannabinol (THC), cannabidiol (CBD), and their acidic precursors (Δ-9-tetrahydrocannabinolic acid A—THCA and cannabidiolic acid—CBDA) at different proportions, together with other minor phytocannabinoids and terpenoids. [1,2,3,4]. Indeed, all these components have been proved essential for anti-inflammatory and neuroprotective properties of the effects of medical cannabis in the symptomatic treatment of neurogenic pain and several other pathologies [5,6].

We recently investigated the disposition of THC, CBD, their precursors, and their principal metabolites in biological matrices of healthy individuals treated with oral decoction and oil, the most used medical cannabis herbal preparations [7,8]. We found that the aqueous preparation was generally better absorbed than the oil, even if it contained a minor amount of THC, THCA-A, and CBD. Consequently, decoction induced a greater feeling of hunger and drowsiness than the oil preparation [7]. In any case, great interindividual variability in cannabinoids kinetics and physiological and subjective effects was observed, especially due to the difficult standardization of herbal preparations and low absorption of oral administrations [9,10,11,12], as previously reported in different studies on adult and young patients administered with oral medical cannabis [13,14].

Conversely, inhalation by vaporization is a promising application mode for medical cannabis due to the very rapid and good absorption of cannabinoids after intrapulmonary administration [15,16]. Different instruments for vaporization have been validated and applied with preliminary presented data providing optimal efficiency of delivery [17].

**Figure 1:**
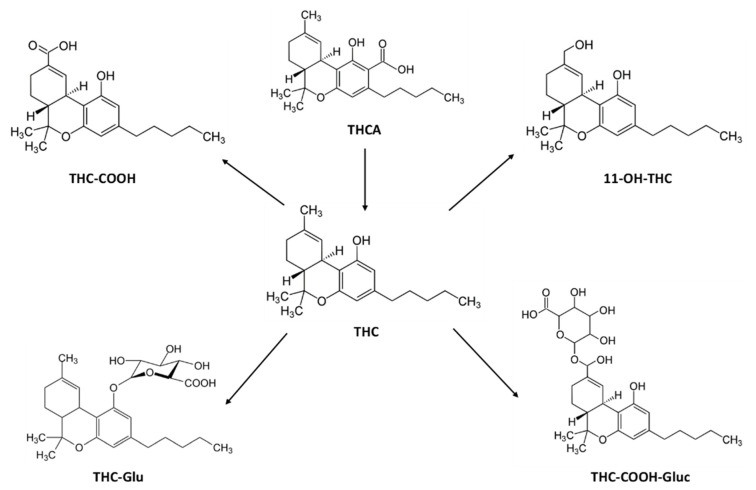
Chemical structures of Δ9-tetrahydrocannabinol (THC), its acidic precursor, and its metabolites.

**Figure 2:**
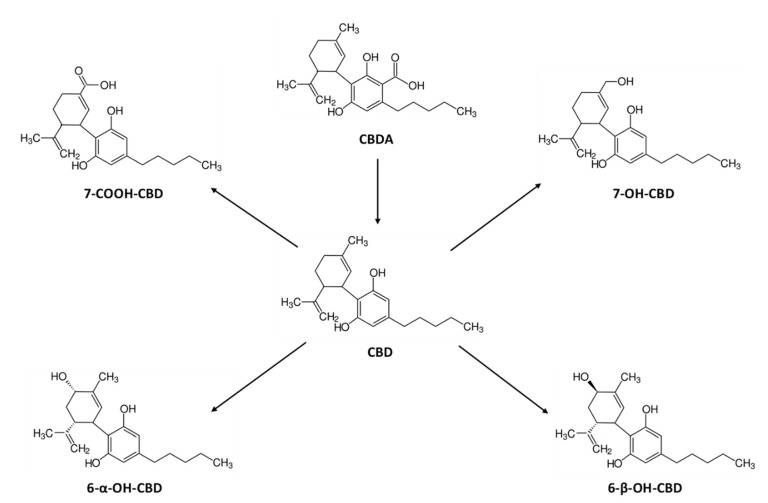
Chemical structures of cannabidiol (CBD), its acidic precursor, and its metabolites.

Taking into account the above-reported evidence, we investigated the disposition and pharmacokinetics of THC, CBD, their acid precursors THCA and CBDA, their principal metabolites: 11-nor-9-carboxy-THC (THC-COOH), 11-hydroxy-THC (11-OH-THC), Δ-9-THC-Glucuronide (THC-GLUC) and THC–COOH–Glucuronide (THC–COOH–GLUC) and CBD-7-oic acid (7-COOH-CBD), 7-hydroxy-hydroxyCBD (7-OH-CBD), 6-alpha-hydroxyCBD (6-α-OH-CBD) and 6-beta-hydroxyCBD (6-β -OH-CBD) (Figure 1 and Figure 2) in serum, oral fluid, and urine samples and acute pharmacological effects of individuals administered with vaporized medical cannabis.

## 2. Results

### 2.1. Subjects and Study Design

All participants completed the study with the exclusion of one male presenting difficulties in coordinating inhalation leading to less than 25% vapor absorption. For this reason, 14 subjects, 12 men and 2 women were finally evaluated.

The 12 males presented a mean age of 21.6 ± 1.0 years (range 20–24 years); mean weight of 69.6 ± 6.3 kg (range 58–85.5 kg), and mean height of 1.8 ± 0.7 m (range 1.7–1.9 m). The 2 females were both 23 years old; weighed 59.1 and 62.0 kg, and were 1.6 and 1.70 m high, respectively. The average onset of cannabis use was 16.1 ± 1.9 years (range 14–18 years), with a mean of 109 cannabis smoking sessions per year (range of 15–320 sessions) in the previous year. In addition, 86% participants reported previous use of oral cannabis (cookies, brownies) and flowering tops smoking through the use of pipes, hookahs, or a bong. All the participants showed previous experience with tobacco, and 64.8% of subjects were regular smokers and alcohol users at the time of the study, with a mean daily use of 1.2 ± 0.7 standard alcohol units (10 g alcohol)/day. Half of the participants had a past history of occasional hallucinogenic mushrooms or truffle consumption and 58% of different psychostimulants (such as ecstasy-MDMA, amphetamines, and cocaine).

The 14 subjects were administered 100 mg FM2 cannabis dried flowering tops containing around 6 mg of total THC (also considering the acidic precursor THCA) and 8 mg of total CBD (also considering the acidic precursor CBDA) vaporized by the Volcano vaporizer. Calculating an inhalation bioavailability of approximately 10–30% [18], the systemically available amount of THC could be considered from 0.6 to 2 mg and CBD from 0.8 to 3 mg.

### 2.2. Concentration-Time Profiles and Pharmacokinetics of THC, CBD, and Their Acidic Precursors in Serum and Oral Fluid after Vaporized Cannabis Administration

**Figure 3:**
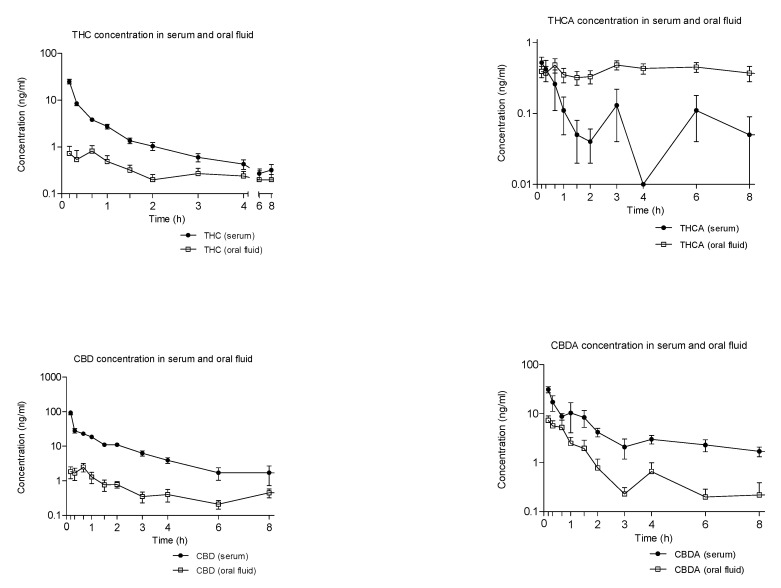
Mean time-course of THC, CBD, and their acidic precursors (THCA and CBDA) concentrations (logarithmic scale) in serum and oral fluid following the administration of vaporized cannabis.

Figure 3 shows the mean time-course of THC, CBD, THCA, and CBDA concentrations in serum and oral fluid following vaporized cannabis administration. Neither THC or CBD metabolites could be detected in this latter biological matrix.

THC peaked firstly in serum and then in oral fluid, with concentrations 20 times higher in the first biological matrices and consequent higher AUCs (Figure 3 and Table 1 and Table 2). In addition, THC acidic precursor THCA firstly peaked in serum and then in oral fluid, although maximum reached concentrations were similar in both matrices. Interestingly, THCA-A-concentrations did not decline in oral fluid up to 6 h post-administration, thus that AUCs in this matrix were 4 times higher than those in serum.

CBD disposition in serum and oral fluid was superimposable to that of THC with concentrations higher than those of THC, due to its superior percentage in medical cannabis (Table 1 and Table 2). CBDA kinetic profile in the above-reported biological matrices was similar to that of CDB, with serum concentrations one-third of those of CBD and oral fluid ones, three times those of CBD (Figure 3 and Table 1 and Table 2).

### 2.3. Concentration-Time Profiles and Pharmacokinetics of THC Metabolites in Seum after Vaporized Cannabis Administration

All THC metabolites showed a later onset with respect to the parent compound in the absorption phase and a slower decrease to baseline.

**Figure 4:**
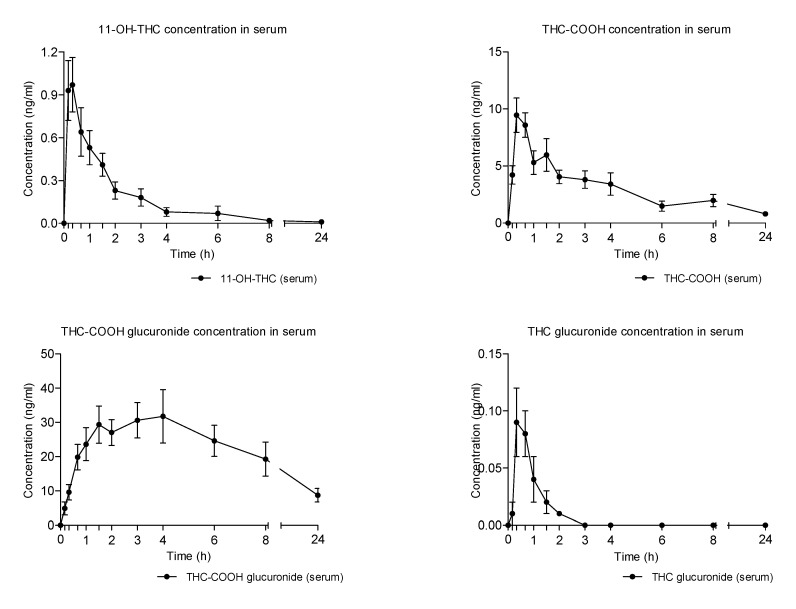
Mean time-course of THC metabolites concentrations in serum following the administration of vaporized cannabis.

This was apparent, especially in the case of THC-COOH-GLUC, which showed the highest serum concentrations among the detected metabolites and a protracted elimination phase during the 24 h time of collection (Figure 4 and Table 2). THC-COOH was the second most abundant THC metabolite, although with AUCs almost 10 times lower than those of THC-COOH-GLUC. Conversely, 11-OH-THC and THC-GLUC presented the lowest serum concentrations and a faster elimination from this biological matrix (Figure 2 and Table 2).

### 2.4. Concentration-Time Profiles and Pharmacokinetics of CBD Metabolites in Serum after Vaporized Cannabis Administration

**Figure 5:**
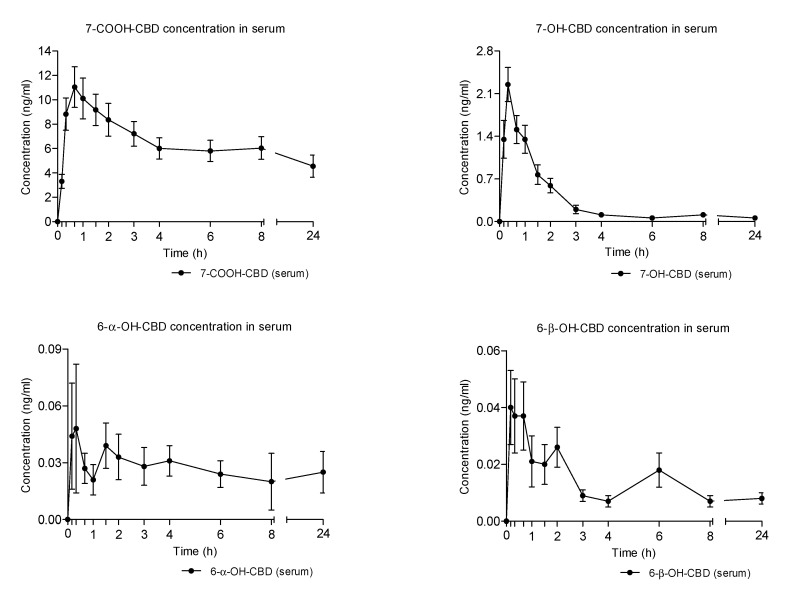
Mean time-course of CBD metabolites concentrations in serum following the administration of vaporized cannabis.

Similarly to what was observed in the case of THC metabolites, CBD metabolites exhibited a delayed onset and decreased to baseline in relation to those of the parent cannabinoid (Figure 5 and Table 1).

The carboxy metabolite, 7-COOH-CBD, showed the highest serum concentrations among the detected metabolites and a protracted elimination phase during the 24 h time of collection (Figure 3 and Table 1). 7-OH-CBD was the second most abundant CBD metabolite with AUCs more than 20 times lower than those of 7-COOH-CBD. Finally, 6-α-OH-CBD and 6-β-OH-CBD presented the lowest serum concentrations (Figure 3 and Table 1).

### 2.5. Urinary Excretion of THC and CBD Metabolites after Vaporized Cannabis Administration

**Figure 6:**
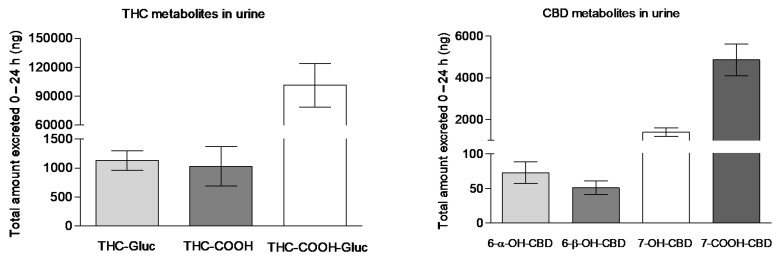
The total amount of THC-GLUC, THC-COOH, THC-COOH-GLUC, and 6-α-OH-CBD, 6-β -OH-CBD, 7-OH-CBD, 7-COOH-CBD excreted in 24 h urine samples following the administration of vaporized medical cannabis.

Following the administration of vaporized medical cannabis, only THC-COOH-GLUC, THC-GLUC, and THC-COOH could be detected in urine, whereas 11-OH-THC was not detected in urine samples as it is mainly excreted in stool and only present at low concentrations in urine as a glucuronide conjugate, whose reference standard was not currently commercially available (Figure 6). In agreement with greater systemic bioavailability, THC-COOH-GLUC was the significantly most excreted THC metabolite. Likely, in agreement with serum time course, 7-COOH-CBD was the highly eliminated CBD metabolite followed by 7-OH-CBD and by minimal amounts of 6-α-OH-CBD and 6-β-OH-CBD (Figure 6).

### 2.6. Physiological Measures and Subjective Effects

**Figure 7:**
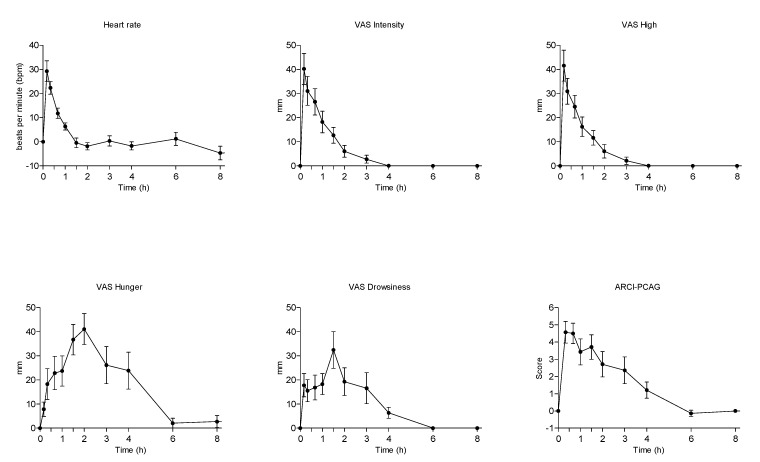
Mean time-course of physiological and subjective mostly modified effects in healthy volunteers administered with 100 mg vaporized cannabis.

The administration of vaporized medical cannabis induced prototypical effects associated with the administration of cannabis or THC in humans (Table 3 and Figure 4) [19,20]. Very mild changes in blood pressure and a significant increase in heart rate, with a peak effect of +25 beats per min were observed at 10 min and persisted at 20 and 40 min post-administration were observed. The scores of some visual analog scales (VAS) feelings of wellbeing as intensity, high, and good effects during almost 1–2 h, increased with peak effects at 10 min post-inhalation. Similar increases in the euphoria-ARCI-MBG scale and amphetamine life effects-ARCI-A were detected 2 h post-administration. Somnolence increased significantly during 3 h with a peak effect of 43 mm in the drowsiness-VAS scale and in the sedation-ARCI-PCAG scale. Hunger sensation increased from 20 min to 4 h. Other effects appeared only at one or two evaluation times (Table 3 and Figure 7). Whereas time courses of hearth rate, feelings of intensity, and high overlapped those of THC and CBD, variations in feelings of hunger, drowsiness, euphoria, and amphetamine-like effects presented a slower onset and a slower decrease to baseline similar to that of THC and CBD metabolites No psychotic effects or other serious side effects were observed.

## 3. Discussion

This is the first study on disposition THC, CBD, their acidic precursors, and their metabolites in serum, oral fluid, and urine samples of healthy adult volunteers after controlled administration of vaporized medical cannabis containing THC and CBD in similar percentages.

As a matter of fact, several previous studies investigated cannabinoids disposition in blood, oral fluid, thus as urinary excretion of metabolites following vaporized cannabis administration, but the cannabis strain selected for considered trials was the recreational one, mainly containing THC at percentages ranging from 2.9% to 6.7% and negligible amounts of CBD [19,20,21,22]. Nevertheless, results obtained for THC and its metabolites are in agreement with those below summarized.

This experiment with vaporized medical cannabis has been the third part of a study on the disposition of THC, CBD, their acidic precursors, and their metabolites following the administration of the three most common formulations of medical cannabis: Decoction, oil, and vaporized flowering tops [7,8]. In this regard, it is worth noticing that:

- Pharmacokinetics of cannabinoids, their precursors, and their metabolites in biological fluids of healthy individuals treated with the three different formulations all showed great interindividual variability, likely due to the difficult standardization of herbal preparations in the case of decoction and oil and different inhalation rates in case of the vaporized formulation;
- A 60 times higher bioavailability and a significantly faster concentration peak of THC was observed in the case of medical cannabis inhalation vs. oral formulations, corresponding to a more than 60 times lower availability of THCA, due to acidic precursor decarboxylation during the vaporization process;
- The proportion between THC metabolites was similar in the three different preparations, with THC-COOH-GLUC being always the most formed metabolite followed by THC-CCOH.
- Oral fluid did not appear a suitable biological matrix alternative to serum for cannabinoids monitoring following vaporized medical cannabis administration since oral fluid concentrations did not reflect the ones measured in serum.
- A more than 90 times higher bioavailability and a significantly faster concentration peak of CBD was observed in the case of medical cannabis inhalation vs. oral formulations, corresponding to a more than 3 times lower availability of CBDA in comparison with that of cannabis decoction due to acidic precursor decarboxylation during the vaporization process. Conversely, the bioavailability of CBDA in cannabis oil as similar to that of vaporized cannabis.
- Whereas minimal salivary excretion did not discriminate different methods of medical cannabis administration, urinary extraction of both THC and CBD metabolites reflected the different parent compound’s availability following the three kinds of medical cannabis formulations.

Our results are quite comparable to those observed in a previous study, in which 200 mg of medical cannabis containing 6.6% THC and 8% CBD were administered to relieve chronic pain in patients with fibromyalgia [23]. THC and CBD C_max_ of 76 and 80 ng/mL, respectively, was found in comparison to 24.92 and 93.17 ng/mL obtained in our study, in which medical cannabis dose was half. With respect to pharmacological effects, an E_max_ in a high feeling of 45 mm at 30 min in the previous study reflected our maximum score of 43.79 mm at 20 min.

It has to be said that the present study shows some limitations: The inclusion of healthy subjects instead of treated patients, the relatively low dose administered in comparison to those recommended for medical cannabis administration, the difficult interpretation of the subjective effects since a placebo condition was not administered, and the fact that the volunteers were almost exclusively males, preventing the evaluation of possible differences between genders.

## 4. Materials and Method

### 4.1. Subjects Enrolment

Fifteen subjects, 13 men and 2 women, were initially recruited. The subjects were recruited by the “word of mouth” method and the database of volunteers who had participated in previous studies at the Clinical Pharmacology Unit, Hospital Universitari Germans Trias i Pujol, Badalona, Spain. The participants were informed of the characteristics of the study and signed informed consent. Eligibility criteria included social or recreational cannabis use in the past 12 months (monthly to weekly use, without criteria of cannabis use disorder) and vaporized cannabis use at least once in their life. Exclusion criteria were history of any serious medical or psychopathological conditions, including substance use disorder (with the exception of nicotine, based on diagnosis and statistical criteria for DSM-5 mental disorders), pregnancy and lactation, chronic medicines use, and serious adverse reactions associated with cannabis use.

Within 3 weeks prior to inclusion in the study, participants underwent a general medical examination, which included blood tests, urinalysis, and a 12-lead electrocardiogram (ECG). In addition, they completed a training session to become familiar with the study procedures and questionnaires. The study protocol was approved by the local Human Research Ethics Committee (CEIC-HGTiP, Badalona, Spain), and the study was carried out in accordance with the Declaration of Helsinki and Spanish laws on clinical research.

### 4.2. Preparation and Inhalation Procedure for Vaporized Cannabis

A standardized medicinal cannabis FM2 flower cup extract containing 3.4% ± 0.7% THC, 2.8% ± 0.8% THCA-A, 2.7% ± 0.5% CBD, and 6.2% ± 1.1% CBDA (plus minor phytocannabinoids with percentages below 0.5%) was provided by the Italian Military Pharmaceutical Chemical Factory in Florence with the authorization of the Italian and Spanish Medicines Agencies [1,2]. Considering that at high temperatures, the acidic precursors THCA and CBDA are decarboxylated to THC and CBD, the total content of these latter compounds can be calculated adjusting for the differing molecular weight of the cannabinoids and carboxylic conjugative components of each cannabinoid:

total % THC: %THC + (0.877%*THCA) = 5.8%

total % CBD: %CBD + (0.877%*CBDA) = 8.1%

The FM2 preparation was administered by inhalation/vaporization using the Volcano vaporizer. The preparation of vaporized cannabis was carried out in accordance with the recommendations of the Volcano Vaporizer System (https://keytocannabis.com/volcano-classic-vaporizer-review-how-to-use-it-and-why-you-need-one/).

For the preparation of inhaled vaporized cannabis, a 100 mg dose of FM2 cannabis administered via the Volcano vaporizer (containing around 6 mg of THC and 8 mg of CBD) was used. Calculating an inhalation bioavailability of approximately 10–30% [24], the available dose exposure dose would be from 0.6 to 2 mg THC and from 0.8 to 3 mg CBD. The procedure for cannabis administration consisted of weighing 100 mg of FM2 cannabis and placing it in the filling chamber. At that point, the vaporizer was set at 225 °C, and the pump was turned on. After the plant material was heated, the valve balloon was fitted, and steam was allowed to flow into the valve balloon for approximately 45 s.

For the vaporized cannabis inhalation, participants had to place their lips on the mouthpiece of the balloon and to inhale the vapor through a deep breath of about 5 s, hold their breath for 5–10 s before exhaling it for an additional 5 s. The subjects then rested for about 10 s and the above-mentioned process was repeated every 30 s until the vapor contained in the balloon ended. Theoretically, participants made an average of 20 inhalations in about 10 min. During this procedure, the subjects remained seated in a chair and could not speak.

### 4.3. Study Design

The study was open-label, non-randomized, and single-blind since the participants knew they would be administered with medical cannabis, but they were not aware of administered doses. The subjects entered the Clinical Research Unit (UPIC) at Hospital Universitari Germans Trias i Pujol, Badalona, Spain at approximately 13:00 h (01:00 p.m.). Upon arrival, participants were asked about any substance/drug or event that could have affected their participation in the study. Subjects had been fasting from food for the previous 3 h, from water for the previous 2 h, in addition to abstinence from alcohol for the preceding 48 h, from coffee, tea, cola, cacao for the previous 24 h, from tobacco smoking for 1 h before. Most importantly, they had not used cannabis from 5 days before the start of the experimental session up to 24 h nor any other psychotropic drug from the screening visit to the end of the study.

Before starting the experimental sessions, a urine sample was taken for the on-site drug test (Instant-View^®^, Multipanel 10 Test Drug Screen, Alfa Scientific Designs Inc., Poway, CA, USA), and alcohol was tested in exhaled air (Dräger alcotest 5820, Dräger, Germany). If a participant resulted positive for drugs or alcohol, he/she was excluded from the experiment. The participants stayed in a quiet and comfortable room in the Hospital’s Clinical Research Unit (UPIC) throughout the session. Tobacco smoking was not allowed. Vaporized cannabis was administered between 14:00 and 14:30 h (02:00–2:30 p.m.), and the experimental session had a duration of 8 h following administration. At the end of cannabis inhalation, the participants’ mouth was washed twice with water to avoid oral contamination in the oral fluid collection. The participants received the treatment with 100 mL of bottled water. Two and 5 h after the administration, subjects received a snack and a meal, respectively. After 4–7 days of each experimental session, a final medical control including physical examination, vital signs, electrocardiogram, blood and urine tests, and collection of adverse events, was conducted.

### 4.4. Physiological and Subjective Effects Measurement

Non-invasive systolic blood pressure (SBP), diastolic blood pressure (DBP), heart rate (HR), oral temperature (T), and subjective effects were repeatedly recorded 45 and 15 min before and 10, 20, 40 min and 1, 1.5, 2, 3, 4, 6, 8, and 24 h after vaporized cannabis administration. Physiological parameters were monitored using a vital sign monitor (Philips SureSigns VM4 monitors, Phillips). Subjective pharmacological effects were measured using the visual analog scales (VAS), the 49-item-short form of the Addiction Research Center Inventory (ARCI), the valuation of the Subjective Effects of Substances with Abuse Potential (VESSPA-SSE) questionnaires, and the Positive and Negative Syndrome Scale (PANSS). The VAS scales used were a set of 15 items (100 mm, from “not at all” to “extremely”) to rate the intensity of the following: Intensity; high; good effects; bad effects; appetite (hunger); drowsiness; dizziness; confusion; nausea; vomiting; anxiety; aggressiveness; visual hallucinations with lights and spots; visual hallucinations with animals, things, insects, or people; auditory hallucinations. The VAS scales were assessed at −45 (baseline), 10, 20, 40 min, and at 1, 1.5, 2, 3, 4, 6, 8, and 24 h after administration.

The ARCI is a true/false 49-item questionnaire, which is a sensitive instrument for determining subjective drug effects and consists of 5 subscales (ARCI-PCAG-sedation, ARCI-MBG-euphoria, ARCI-LSD- dysphoria and somatic symptoms, ARCI-BG-intellectual efficiency, ARCI-A-amphetamine-like effect) [25,26]. This was administered at the same times as VAS, except for the 10 min evaluation. The VESSPA-SE questionnaire measured changes in subjective effects caused by a number of drugs. It included 6 subscales: Sedation (S), psychosomatic anxiety (ANX), changes in perception (CP), pleasure and sociability (SOC), activity and energy (ACT), and psychotic symptoms (PS) [27,28]. It was administered at −45 min (baseline) and at 1, 2, and 8 h after administration. The PANSS for the evaluation of psychotic symptoms was scored before and 8 h after substance administration.

### 4.5. Biological Samples Collection

Serum (2 mL) from whole blood was collected from 15 min (zero time) prior and 10 min, 20 min, 40 min, 1, 1.5, 2, 3, 4, 6, 8, and 24 h after vaporized cannabis administration. Oral fluid samples were collected using a Salivette device (Sarsted, Nümbrecht, Germany) at the same time points. Before oral fluid collection, participants had to wash their mouth with two glasses of water. Urine was collected 15 min prior to administration (baseline time) and then between 0–2 h, 2–4 h, 6–8 h, and 8–24 h intervals. After collection, all biological samples were stored at −30 °C until analysis.

### 4.6. Determination of Cannabinoids, Acidic Precursors, and Metabolites in Biological Samples

THCA, THC, THC-COOH, 11-OH-THC, THC-GLUC, THC–COOH–GLUC, CBDA, and CBD were quantified in the collected biological matrices by ultrahigh-performance liquid chromatography-tandem mass spectrometry (UHPLC-MS/MS) equipped with an electrospray ionization source operating in positive ion mode as previously reported [29]. With respect to CBD metabolites, 6-α-OH-CBD, 6-β -OH-CBD, 7-OH-CBD, 7-COOH-CBD, a specific UHPLC-MS-MS assay recently developed and validated [30]. For both assays, limits of quantification ranged from 0.05 to 0.2 ng/mL. with average inter/intra-day accuracy and precision <15% [29,30].

### 4.7. Statistical Analysis

Maximum concentration (C_max_), time needed to reach maximum concentrations (T_max_), area under the concentration-time curve at 10 and 24 h (AUC_0–10h_, AUC_0–24h_), elimination half-life (t_1/2_), elimination constant (K_e_, calculated with at least 3 sample points), mean residence time (MRT as the area under the concentration times time versus time curve-AUMC_0–24h_ divided by AUC_0–24h_) for THC, CBD, their acidic precursors, and their metabolites in serum and oral fluid were determined using Pharmacokinetic Functions for Microsoft Excel (Joel Usansky, Atul Desai, and Diane Tang-Liu, Department of Pharmacokinetics and Drug Metabolism, Allergan, Irvine, CA, USA at https://www.coursehero.com/file/30859156/pkfdoc/).

Maximum effects (Emax) were determined and the areas under the curve of the effects (AUC_0–8h_) were calculated using the trapezoidal rule. To evaluate the physiological (SBP, DBP, HR, and T) and subjective effects (VAS, ARCI, VESSPA) along time in comparison to baseline, a one-way repeated measures ANOVA, with time as a factor, was done to evaluate the time-course of the effects. When the time condition was statistically significant, a Dunnett multiple comparison post hoc test was conducted to compare the different time points with baseline.

## 5. Conclusions

Notwithstanding the listed limitations, the results obtained in this study are relevant and in agreement with those already presented on the issue of oral cannabis preparations: The pharmacokinetics of cannabinoids, their precursors, and their metabolites in biological fluids of individuals treated with medical cannabis preparations showed high interindividual variability, especially due to the difficult standardization of herbal preparations. Vaporized medical cannabis was earlier absorbed into the organism, cannabinoids reached higher systemic concentrations, also due to the fact that the acid precursors decarboxylated to parent cannabinoids at high temperatures. Consequently, the physiological and subjective effects occurred earlier and were higher. In no case did adverse effects appear, although the doses administered had always been low. Greater clinical trials, including both healthy volunteers and patients suffering from pathologies treated with medical cannabis and a larger number of women, and studying clinical and side effects of chronic administration will complement these significant preliminary observations.

## Figures and Tables

**Table 1:** Pharmacokinetic parameters of THC, CBD, and their acidic precursors in oral fluid of healthy volunteers administered with 100 mg vaporized medical cannabis.

	Pharmacokinetics Parameters (mean ± SD)			
	C_max_ (ng/mL)	T_max_ (hour)	AUC_0__–__8h_ (ng/mL·h)	AUC_0__–__24h_ (ng/mL·h)
THC	1.29 ± 1.11	0.67 (0.17–24.0)	2.26 ± 2.32	4.90 ± 5.22
THCA	0.78 ± 0.26	3.50 (0.17–8.0)	3.26 ± 1.44	7.79 ± 4.29
CBD	3.27 ± 2.77	0.67 (0.17–8.0)	4.88 ± 3.90	11.36 ± 8.88
CBDA	10.22 ± 8.49	0.17 (0.17–1.5)	8.79 ± 6.87	13.17 ± 12.60

C_max_, maximum concentration; T_max_, time needed to reach maximum concentration; AUC_0–8h_ and AUC_0–24h_, area under the concentration–time curve at 18 and 24 h; SD, standard deviation.

**Table 2:** Pharmacokinetic parameters of THC, CBD, acidic precursors, and metabolites in serum of healthy volunteers administered with 100 mg vaporized medical cannabis.

	Pharmacokinetics Parameters (mean ± SD)						
	C_max_ (ng/mL)	T_max_ (hour)	AUC_0–8h_ (ng/mL·h)	AUC_0–24h_ (ng/mL·h)	K_e_ (h^−1^)	t_1/2_ (hour)	MRT (hour)
THC	24.92 ± 10.74	0.17	12.21 ± 4.11	15.91 ± 6.76	0.79 ± 0.56	1.75 ± 1.97	3.59 ± 4.06
THCA	0.95 ± 0.47	0.33 (0.17–8.0)	0.80 ± 0.1	1.49 ± 2.93	ND	ND	3.37 ± 2.17
11-OH-THC	1.43 ± 0.66	0.33 (0.17–1.0)	1.67 ± 0.99	1.91 ± 1.17	0.83 ± 0.79	1.25 ± 0.65	3.05 ± 2.75
THC-COOH	12.08 ± 4.76	0.50 (0.33–2.0)	28.05 ± 16.71	50.23 ± 35.02	ND	ND	6.73 ± 2.16
THC-COOH-GLUC	41.85 ± 26.81	3.0 (1.5–8.0)	213.83 ± 132.38	450.52 ± 322.36	0.06 ± 0.02	12.62 ± 3.79	8.55 ± 0.73
THC-GLUC	0.12 ± 0.11	0.5 (0.0–1.5)	0.09 ± 0.08	0.10 ± 0.09	ND	ND	1.4 ± 1.14
CBD	93.17 ± 44.77	0.17	68.84 ± 18.25	88.42 ± 50.53	0.46 ± 0.30	3.45 ± 4.72	3.12 ± 2.68
CBDA	34.54 ± 21.54	0.17 (0.17–3.0)	36.90 ± 20.52	63.80 ± 23.21	1.02 ± 1.15	4.75 ± 7.29	7.76 ± 4.18
7-COOH-CBD	11.92 ± 6.29	0.67 (0.67–3.0)	55.38 ± 28.65	140.17 ± 78.63	ND	ND	10.23 ± 1.83
7-OH-CBD	2.36 ± 1.02	0.33 (0.17–1.5)	3.28 ± 1.81	4.62 ± 2.82	0.77 ± 0.63	3.22 ± 5.10	4.71 ± 1.90
6α-OH-CBD	0.11 ± 0.11	1.75 (0.17–6.0)	0.22 ± 0.18	0.59 ± 0.94	ND	ND	1.00 ± 0.00
6β-OH-CBD	0.08 ± 0.05	0.67 (0.17–24.0)	0.13 ± 0.09	0.25 ± 0.14	ND	ND	8.80 ± 4.28

SD, standard deviation; C_max_, maximum concentration; T_max_, time needed to reach maximum concentration (hour, range); AUC_0–8h_ and AU_0–24h_, area under the concentration–time curve at 8 and 24 h; K_e_, elimination constant; t_1/2_, elimination half-life. MRT, mean residence time from 0–24 h. ND: Not done. Pharmacokinetic parameters could not be calculated since the number of decreasing points to calculate these parameters was insufficient (less than 3).

**Table 3:** Physiological and subjective effects in healthy volunteers administered with 100 mg vaporized medical cannabis.

Effects	E_max_ (mean ± SD)	T_max_ (Median-Range)	AUC_0–8h_ (mean ± SD)
Systolic blood pressure, mm Hg	−6.57 ± 16.37	1.34 (0.17–6.0)	−46.14 ± 54.18
Diastolic blood pressure, mm Hg	2.93 ± 14.12	0.84 (0.17–6.0)	−16.04 ± 46.15
Heart rate, bpm	25.64 ± 23.23	0.17 (0.17–8.0)	10.97 ± 55.62
VAS Intensity, mm	42.86 ± 22.45	0.17 (0.17–0.67)	44.20 ± 35.69
VAS High, mm	43.79 ± 22.87	0.17 (0.17–0.33)	42.18 ± 32.15
VAS Good effects, mm	33.00 ± 22.38	0.33 (0.0–1.0)	37.34 ± 30.49
VAS Bad effects, mm	13.00 ± 19.42	0.17 (0.0–0.33)	7.14 ± 12.94
VAS Appetite, mm	47.36 ± 27.42	2.0 (0.0–3.0)	141.31 ± 113.40
VAS Drowsiness, mm	39.00 ± 28.25	1.5 (0.17–3.0)	76.83 ± 59.86
VAS Dizziness, mm	19.64 ± 22.38	0.17 (0.0–1.0)	10.06 ± 13.36
VAS Confusion, mm	11.79 ± 16.04	0.17 (0.0–0.67)	4.27 ± 5.90
VAS Nausea, mm	1.57 ± 4.85	0.0 (0.0–0.67)	0.80 ± 2.80
VAS Vomit, mm	1.43 ± 5.35	0.0 (0.0–0.33)	0.46 ± 1.72
VAS Anxiety, mm	7.0 ± 14.82	0.0 (0.0–0.33)	4.66 ± 10.86
ARCI-PCAG, score	5.71 ± 2.02	0.33 (0.33–3.0)	12.25 ± 8.82
ARCI-MBG, score	4.21 ± 4.58	0.33 (0.0–2.0)	8.26 ± 11.61
ARCI-LSD, score	2.14 ± 2.54	0.33 (0.33–1.5)	0.81 ± 3.46
ARCI-BG, score	−1.00 ± 2.57	0.67 (0.33–4.0)	0.03 ± 5.54
ARCI-A, score	3.00 ± 2.39	0.33 (0.0–1.5)	7.63 ± 8.45
VESSPA-S, score	0.90 ± 0.53	1.0 (1.0)	2.88 ± 2.11
VESSPA-SA, score	0.69 ± 0.31	1.0 (1.0–2.0)	1.86 ± 1.17
VESSPA-CP, score	0.02 ± 0.09	0.0 (0.0–1.0)	0.02 ± 0.09
VESSPA-PCS, score	0.64 ± 1.08	1.0 (0.0–2.0)	2.69 ± 4.75
VESSPA-AE, score	0.36 ± 0.65	0.0 (0.0–8.0)	1.19 ± 2.35
VESSPA-SP, score	0.46 ± 0.75	1.0 (0.0–2.0)	0.83 ± 0.99

E_max_ = peak effects; T_max_ = time needed to reach maximum effects; AUC_0__–8h_ = area under the curve from 0 to 8 h; SD (standard deviation); VAS (Visual analog scales); ARCI (Addiction Research Center Inventory); VESSPA-SEE (Evaluation of subjective effects of substances with abuse potential).

## Data Availability

The data presented in this study are available within the article or on request from the corresponding author.
